# Our New York Letter

**Published:** 1891-01

**Authors:** Wm. L. Russell

**Affiliations:** New York


### Correspondence.

### OUR NEW YORK LETTER.

New York, December 15, 1890.

### KOCH'S REMEDY.

Interest in Koch's discovery has been intensified here during the past week, by the reception of some of the "lymph," as it is called, in this city and New Haven. It was somewhat of a surprise to learn that the city of New Haven had taken the precedence in the introduction of the remedy into this country, and that the first experiments were to be made by a comparatively unknown practitioner. The circumstances of the case are said to be as follows: A Mr. Blake, of New Haven, had a son who was suffering from phthisis. When he read in the papers about the wonderful remedy which had been discovered in Berlin, he went to his friend, Prof. Chittenden, of Yale College, and induced him to try and secure some of it to use on his son. The latter communicated with an intimate friend, a professor in Heidelberg University, and through him a small quantity was soon obtained. Prof. Chittenden, not being a practicing physician, handed it over to the physician of the young man, Dr. Foster, who, with Dr. Francis Bacon, immediately began a series of experiments. The results have not yet been officially announced.

Experiments in New York began early last week, and now quite a large number of cases of all forms of tubercular disease have been inoculated. The first injections were made at the Hospital for Ruptured and Crippled, by Dr. Allen McLane Hamilton. A number of cases are also under treatment at St. Luke's by Dr. Kinnicutt, and a still larger number at Mt. Sinai by Prof. Jacobi. It is too soon to give any particulars regarding the results of these experiments, but so far as I can learn the symptoms following the inoculations have differed in no particular from those observed in Berlin, and referred to in your last issue. Cases of lupus, tubercular joint and bone lesions, tubercular glands, and pulmonary phthisis are among those under treatment, and a very satisfactory investigation of the subject may be expected.

The reports which reach us from Berlin are nearly all of the same character, and tend to confirm the claims made by Koch. The efficacy of the remedy in cases in which the tubercular lesion is visible is said to be absolutely proved. Its effect, however, is to localize the disease rather than to remove it. The bacilli are not destroyed, as Koch expected would be the case, but the tubercular tissue is separated from the healthy by a limiting wall of new tissue. Whether such a process will result in the cure of tubercular lung disease cannot yet be decided. It is certain, however, that the disappearance of all symptoms, such as fever, cough and expectoration, hæmoptysis, etc., and restoration to apparent health have been accomplished. This is only in the early stage of the disease, though; advanced cases with cavities are not benefited to any extent, if at all.

A few observers are unable to feel satisfied regarding the efficacy of the remedy. Dr. Saryuski, of Cracow, who was sent to Berlin by the Medical Society of Cracow, has reported that the cure of even external tuberculosis is uncertain, while he sees no grounds for believing that consumption in any stage will be cured. Dr. Stellwag, of Vienna, in addressing the students, said that so far the possibility of curing lupus alone had been proved, while it had not been scientifically established that lupus arose from the same bacillus which was associated with lung tuberculosis.

A case of tuberculosis of the larynx has been reported by Dr. Krause, of Berlin, in which the healing process can be observed daily. The Berlin correspondent of the Medical Record has also had an opportunity of observing a number of cases under treatment in the private hospital of Dr. Levy. He reports that among them was one of tubercular disease of the tarsus, of several months' duration, and having a sinus from which was discharged thin pus. After an injection of the remedy in the usual location, the foot became swollen, red, tender and painful, and the discharge thick and creamy. The usual constitutional symptoms appeared and lasted two or three days. A second injection was followed by a repetition of these symptoms, but not so marked, and a similar result followed the third. After the fourth injection the sinus closed. The fifth and sixth caused no constitutional symptoms, and no local phenomena except a slight redness around the site of the sinus. Neither constitutional nor local symptoms followed the seventh injection, and the case was then pronounced cured.

Quite alarming symptoms have occurred in a few cases. The reaction has been accompanied by great prostration with dyspnœa, requiring the liberal use of stimulants. At the Mt. Sinai Hospital here there have been instances of what appeared to be an accumulative action of the remedy, the dose which one day produced only a moderate reaction, on the next causing the most pronounced symptoms. In a few cases in Berlin, coma resulted, lasting for a short time, and in one case, in which it remained for forty-eight hours, it was feared that the patient would die.

### LIFE-SAVING METHODS IN STILL-BIRTH.

At the last meeting of the Academy, Prof. Lusk read a paper on life-saving methods in still-births. He stated that he had been led to select this subject by a recent experience, which illustrated the difficulties which beset these cases, and the success which patient and thoughtful effort could attain. Three hours of steady effort had been necessary to save the child. He then gave a short review of the physiology of the fœtal circulation, and the changes which took place at birth, and went on to describe his method of treatment.

In the more ordinary cases the indications were to clear the air passages, restore the irritability of the medulla, and relieve the engorgement of the heart and great vessels. The usual plan of clearing out the throat, of mouth to mouth expansion of the lungs, of spanking, and the use of cold and hot water were, as a rule, sufficient to restore the child. These cases, however, often died in a few days of atelectasis. To prevent this he recommended the swinging movements of Schultz. These should be resorted to as soon as the child was restored, and were performed as follows: The operator grasped the child by the shoulders, the thumbs being on the anterior chest wall, and the fingers in the armpits, the head of the child resting between the hands. The body was then swung forwards and upwards at arms' length until the feet swung clear over approximating the head. This should be repeated once or twice. In this way the lungs were completely expanded, and ventilated, the mucus expelled, and the congested heart cavities and great vessels unloaded by compression. Many cases could be saved in this way. It should not be employed, however, if the heart was very feeble.

In cases of deep asphyxia, when the child was pallid and limp, the ordinary methods should be resorted to, but with as little disturbance as possible, and only for a very short time. The child should then be laid on a table, covered warmly, and a number eight English moderately stiff elastic catheter should be passed into the trachea, and suction employed to clear it of fluid. It might be necessary to repeat this manœuvre several times. It was not dangerous. After clearing the trachea, the lungs should be moderately inflated by gently blowing through the catheter, and then as gently emptied by compression of the chest walls. The Sylvester method of artificial respiration should next be employed, care being taken to draw the tongue well forward, and to have an assistant hold the lower extremities. The movements should correspond in number to the normal. Just as soon as the heart impulse could be plainly felt, the face should be sprinkled with warm and cold water alternately, and if the circulation were too feeble the Schultz movement should complete the treatment. Patience, watchfulness and hopefulness were necessary in successfully managing these cases. When, however, the life of the child was saved, there were few victories more satisfactory than this triumph of scientific skill and patient effort.

The paper was discussed by Prof. Polk, Dr. H. J. Garrigues, of Maternity Hospital, Prof. Jacobi and others. All agree with the main points of the paper. One speaker, however, stated that he had found a modification of the Schultz movements very satisfactory. His plan was to hold the child across both hands, the back of the neck resting in the palm of the left hand, the legs being grasped in the right. Then by alternately "doubling up" and extending the child's body, the great vessels were compressed and emptied, the lungs were emptied, and then expanded to their fullest capacity in a manner somewhat similar to that introduced by Schultz, but with a much greater appearance of gentleness.

THE NEW ACADEMY BUILDING.

One of the notable events of the past month was the opening of the new academy building. As stated in the editorial columns of your last issue, the event marked an era in American medicine. It is certainly a matter of congratulation to the whole country that the academy is now better equipped than ever for its great and useful work. The building is on West Forty-third street, and its appearance is in keeping with the purpose for which it is intended. One enters between a pair of massive pillars which might have been taken from some Egyptian temple. The interior is plain almost to severity. It is finished in black oak, and the floors are bare save for a strip of carpet in the halls and in the passages between the seats in the meeting rooms.

A distinguished company gathered at the opening ceremonies. More than a thousand persons were present. Several addresses were delivered, and a characteristic letter from Dr. Oliver Wendell Holmes was read by President Loomis. It states so clearly the true purpose of the academy, that I cannot do better than to quote a portion. After referring to the French and other academies, he says: "But the academy which fulfils its true function is a worklng body. It deals with living subjects; it handles unsettled questions; it sets tasks for its members and furnishes so far as it can the appliances for their prosecution; it offers rewards for meritorious performances, and sits in judgment on the efforts of aspirants for distinction. It furnishes the nearest approach we can expect to a fixed standard of excellence by which the work of new hands and the new work of old hands can be judges. It is a barrier, a breakwater against the rush of false pretensions which are constantly attempting to find their way into public confidence. Nowhere is such a defence more needed than in the sciences and arts which deal with the health of the community. The public is so ready, so eager to be deceived, and the traders in deception are so willing, so hungry to deceive those who will listen to them, that it needs a solid wall of resistance, a close united phalanx of men of recognized sense, knowledge and character to stand against them. The various forms of what I will venture to christen as pseudo-pathy, and pseudo-therapy—though they are known to the public by other names—can loosen the hold of the intelligent thoroughbred physician on the enlightened members of society, so long as the best heads in the profession are banded together in a noble institution like this academy."

SUCCI.

There is some interest manifested in an Italian named Succi, who is attempting a prolonged fast in this city. He has already accomplished nearly forty days, and has not yet suffered from any dangerous symptoms. He is not an educated or scientific man, and fasts only for the purpose of exhibiting himself. He is thirty-eight years old, and usually weighs one hundred and forty-seven pounds. He is very muscular and inclined to be spare, having very little fat on him. He has been twice confined in a hospital for the insane ; he says because of his announcements regarding his fasting powers ; and though he has been examined by several physicians no delusions or extraordinary psychological theories have been discovered. He fasted for thirty days in Italy, about two years ago, and is said not to be an impostor. He takes nothing but plain water and a few drops of an elixir which undoubtedly contains opium. His weight has now been reduced to one hundred and seven pounds, and he has grown somewhat irascible in manner. His urine is nearly normal, and his pulse seldom goes above seventy.

WM. L. RUSSELL.

